# Molecular epidemiology, transmission and clinical features of 2022‐mpox outbreak: A systematic review

**DOI:** 10.1002/hsr2.1603

**Published:** 2023-10-05

**Authors:** Nadim Sharif, Nazmul Sharif, Khalid J. Alzahrani, Ibrahim F. Halawani, Fuad M. Alzahrani, Isabel De la Torre Díez, Vivían Lipari, Miguel Angel López Flores, Anowar K. Parvez, Shuvra K. Dey

**Affiliations:** ^1^ Department of Microbiology Jahangirnagar University Savar Dhaka Bangladesh; ^2^ Department of Mathematics Rajshahi University of Engineering & Technology Rajshahi Bangladesh; ^3^ Department of Clinical Laboratories Sciences, College of Applied Medical Sciences Taif University Taif Saudi Arabia; ^4^ University of Valladolid Valladolid Spain; ^5^ Universidad Europea del Atlántico Santander Spain; ^6^ Universidad Internacional Iberoamericana Arecibo Puerto Rico USA; ^7^ Universidade Internacional do Cuanza Cuito Bié Angola; ^8^ Fundación Universitaria Internacional de Colombia Bogotá Colombia; ^9^ Universidad Internacional Iberoamericana Campeche México; ^10^ Instituto Politécnico Nacional UPIICSA Ciudad de México México

**Keywords:** clinical features, epidemiology, monkeypox, transmission

## Abstract

**Background and Aims:**

The 2022‐mpox outbreak has spread worldwide in a short time. Integrated knowledge of the epidemiology, clinical characteristics, and transmission of mpox are limited. This systematic review of peer‐reviewed articles and gray literature was conducted to shed light on the epidemiology, clinical features, and transmission of 2022‐mpox outbreak.

**Methods:**

We identified 45 peer‐reviewed manuscripts for data analysis. The standards of the Preferred Reporting Items for Systematic Review and Meta‐Analysis (PRISMA) Statement and Cochrane Collaboration were followed for conducting the study.

**Results:**

The case number of mpox has increased about 100 times worldwide. About 99% of the cases in 2022 outbreak was from non‐endemic regions. Men (70%–98% cases) were mostly infected with homosexual and bisexual behavior (30%–60%). The ages of the infected people ranged between 30 and 40 years. The presence of HIV and sexually transmitted infections among 30%–60% of cases were reported. Human‐to‐human transmission via direct contact and different body fluids were involved in the majority of the cases (90%–100%). Lesions in genitals, perianal, and anogenital areas were more prevalent. Unusually, pharyngitis (15%–40%) and proctitis (20%–40%) were more common during 2022 outbreak than pre‐2022 outbreaks. Brincidofovir is approved for the treatment of smallpox by FDA (USA). Two vaccines, including JYNNEOSTM and ACAM2000®, are approved and used for pre‐ and post‐prophylaxis in cases. About 100% of the cases in non‐endemic regions were associated with isolates of IIb clade with a divergence of 0.0018–0.0035. Isolates from B.1 lineage were the most predominant followed by B.1.2 and B.1.10.

**Conclusion:**

This study will add integrated knowledge of the epidemiology, clinical features, and transmission of mpox.

## 1 INTRODUCTION

The 2022 mpox (previously monkeypox) outbreak has transmitted across 110 countries with case number surpassing 86,716 on March 29, 2023.^1^, ^2^ No historical records of mpox have been reported from the majority of the localities (94%, 103 of 110) before the 2022 outbreak.^1^, ^2^, ^3^, ^4^, ^5^ Nearly 99% cases and fatalities are reported from these non‐endemic regions.^1^, ^2^, ^3^ The highest number of cases (nearly 30,000) have been recorded in the United States with 20 deaths.^1^, ^2^, ^3^

Historically, monkeypox virus was isolated in 1958 from outbreaks in cynomolgus monkeys with smallpox‐like symptoms in Copenhagen.^3^, ^4^, ^5^ During 1970s, several outbreaks were reported from monkeys in the United States and the Netherlands. The first symptomatic case of human mpox was documented in 1970 during smallpox surveillance in Democratic Republic of Congo (DRC) from a 9‐month‐old child followed by another six cases in young children during 1970–1971 in West Africa.^3^, ^4^, ^5^

Before the ongoing 2022 outbreak, mpox was considered a rare zoonotic disease.^4^ Monkeypox virus is a double‐stranded DNA virus belonging to the family Poxviridae, subfamily Chordopoxvirinae, and genus *Orthopoxvirus*.^6^ The genus includes other important pathogens of humans and animals, including mpox, cowpox, camelpox, vaccinia, and the smallpox. Symptomatic infection of monkeypox virus results in smallpox‐like symptoms in patients. Further, monkeypox and smallpox viruses are highly similar both in genetic and antigenic properties.^6^, ^7^ The genome of monkeypox virus is about 200 kb in size, linear, and contains hairpin ends with inverted terminal repeats. Nearly 200 proteins are encoded by mpox virus. Housekeeping genes are encoded from the central conserved regions. Antigenic proteins are encoded by the terminal regions of the genome and vary between poxviruses.^6^, ^7^

The animal reservoirs of zoonotic monkeypox virus are still unknown. Further, the intermediate hosts of monkeypox virus are also unknown.^4^, ^6^, ^7^ Apes and monkeys are the most likely intermediate hosts and several rodent species, including tree squirrels, rope squirrels, Gambian pouched rats, and dormice, are the most probable animal reservoir of monkeypox virus.^4^ The natural history of monkeypox virus is yet to be discovered.

Based on the findings of previous sporadic and recent 2022 outbreaks, the transmission route of monkeypox virus can be characterized.^8^, ^9^, ^10^, ^11^, ^12^ The recent findings support the presence of monkeypox virus in semen and man to man sexual transmission.^11^, ^12^, ^13^, ^14^ Transmission of monkeypox virus has been reported via saliva, respiratory droplets, aerosols, and close contact, direct contact with lesions, contaminated fomite, and possibly air.^13^, ^15^, ^16^, ^17^, ^18^, ^19^, ^20^, ^21^ Parenteral transmission and fetal deaths of mpox have been reported.^21^, ^22^

Patients with monkeypox virus infection develop characteristic symptoms.^23^, ^24^ The most common symptoms include fever, multiple popular lesions, vesiculopustular lesions, and ulcerative lesions on the body and face and lymphadenopathy.^4^, ^13^, ^15^, ^16^, ^17^, ^18^, ^19^, ^23^, ^25^, ^26^ Case fatality rate may vary between 1% and 10% depending on the clade of monkeypox virus infection.^4^, ^27^, ^28^, ^29^ Severe illnesses and other complications like encephalitis, pneumonitis, and secondary infections are higher in children, elderly, and HIV‐infected patients.^23^, ^25^, ^27^, ^29^

The discontinuation of routine vaccination of smallpox since 1980 and asymptomatic circulation of monkeypox virus in humans may have contributed significantly to changes of biological properties of the virus.^4^, ^6^, ^7^ Further, changes in human behaviors and movement have probably contributed to the 2022 outbreaks. Studies on the epidemiology, clinical characteristics, and transmission route are scarce. Only few review studies are available with limited knowledge of the previous sporadic outbreaks.^4^, ^6^, ^7^ Recently, as case numbers are increasing, studies focusing on clinical features and transmission are getting highlighted to understand the baseline of the outbreaks. This study was conducted to create integrated insights about the epidemiology, route of transmission in the human body and environment along with clinical data of monkeypox virus.

## 2 METHODS

### 2.1 Definitions

The epidemiology of mpox is defined as the distribution and determinants of outbreaks in different populations and strategies taken to minimize the health effect in different population. The clinical feature was defined, including both the signs and symptoms during and after the mpox infection. Transmission of monkeypox virus was defined as the transfer of the virus from human to human, reservoirs to human, and carrier to human. This study included epidemiological, clinical, and virological studies. The PCR‐positive laboratory‐confirmed test was defined as the positive mpox case. The standards of the Preferred Reporting Items for Systematic Review and Meta‐Analysis (PRISMA) Statement and Cochrane Collaboration were followed for conducting the study.^30^ The study is submitted for registration in the PROSPERO (ID 391380).

### 2.2 Study design

The study was conducted by the following different steps, including identification of precise objectives and search strategies, appropriate research articles, inclusion of manuscripts, collection of data, analysis, and summarization of the findings. This study included previous findings from epidemiological studies, case studies, outbreak investigation, surveillance work, and online databases. No strict parameters for the quality assessment of these studies are available. As a result, this study relied on the quality report of the selected articles by the authors.

### 2.3 Search strategy and selection criteria

We performed searches for articles in MEDLINE (through PubMed), EMBASE, Web of Science, Scopus, the Internet Library sub‐Saharan Africa (ilissAfrica), African Journals Online (AJOL), *The New England Journal of Medicine* (NEJM) and *The Lancet* with no restriction on language and place. All published articles and scientific writings till March 20, 2023 were included in this study. The notable search term included Monkeypox, Monkeypox virus, Monkey pox, MPXV, Epidemiology of mpox, Epidemiology of Monkeypox, Clinical features of monkeypox, Sign and Symptoms of Monkeypox, Clinical characteristics of mpox, Cases of Monkeypox, Transmission of mpox, Transmission of Monkeypox, “variole simienne,” and “variole du singe” and combination of these terms. Separate searches were made for each term in every website and database.

Additionally, we conducted search on the gray literature and Google Scholar. These sources included databases from CDC (Centers for Disease Control and Prevention, USA), ECDC (European Centers for Disease Control and Prevention), WHO (the World Health Organization), Epicentre, ProMed, CDC of Nigeria, CDC of Africa, and African Field Epidemiology Network. We searched for the weekly and monthly updates on the emergencies and update of the data in these websites and databases. Further, we searched and included data from different preprint databases like bioRxiv, SSRN, medRxiv, and AAS Open Research. Additionally, the first 10 pages of the Google Scholar search system for each search term were manually analyzed for relevant articles. We tried to determine the epidemiology of mpox, including transmission dynamics, incidence, case reports, clinical history, case fatality rate, and distribution of clades. Further, we included data identifying the risk factors and risk groups of mpox.

Two authors, N. S. and S. K. D. conducted the evaluation of eligible studies. After completion of the search of all the databases, the potential articles were selected by removing the duplicates and screening by N. S. and K. J. A. Articles with specific and relevant topics were selected for all regions, all ethnicity, age group, sex, and clinical features for full‐text analyzing. We also included the articles focusing on the transmission of mpox involving animals and humans to better understand the mode of transmission. We excluded modelling and prediction studies, studies on smallpox, review articles, and studies nonrelevant to our objectives. Further, critical evaluation of the quality of selected articles, analyzing for duplicated articles, and removing correspondence or comment of duplicated data were performed by N. S., S. K. D., and K. A. J. separately.

The seasonal exclusion criteria could not be implemented due to lack of studies on seasonality and environmental impacts. Furthermore, we could not exclude studies that did not include data on specific clades due to a lack of work.

We used the Systematic Review Centre for Laboratory Animal Experimentation (SYRCLE) assessment tool for measuring the risk of bias.^31^, ^32^ The SYRCLE consists of 10 parameters to assess different biases in studies. The parameters included detection bias, attrition bias, reporting bias, selection bias, performance bias, and other biases. The bias for each parameter was measured by using the possible outcomes as yes, no, and unclear, representing low, high, and unclear bias, respectively.^31^, ^32^

### 2.4 Case definition

A confirmed case of monkeypox virus was defined by following the UK Health Security Agency (UKHSA) definition: a positive result on mpox PCR assay in a specimen collected from any anatomical site is considered as a positive case. The type of the used PCR assay will be determined on the basis of local guidelines and availability of the test method.

### 2.5 Statistical analysis

A total number of cases was determined for each decade by summation of the reported cases per clade. Pooled Statistical analyses were conducted by using SAS version 9.4.

## 3 RESULTS

### 3.1 Studies included

**Figure 1:**
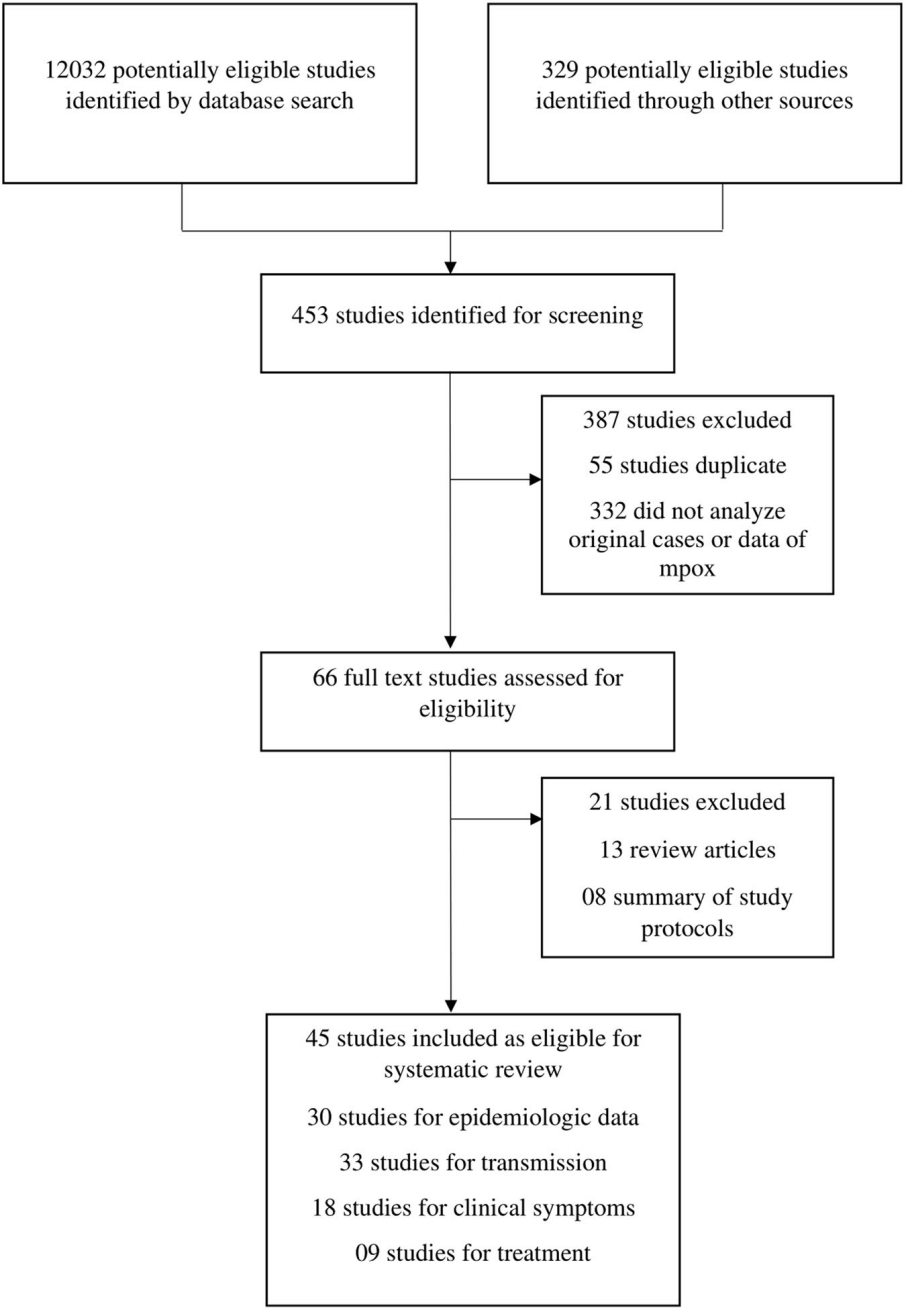
Selection procedure of peer‐reviewed articles. The excluded articles were duplicate, reviews, correspondence, editorial, and failed to meet inclusion criteria.

This study detected 12,032 research articles on epidemiology and transmission of monkeypox virus and previously mentioned related search terms (Figure 1). Among 12,032 articles, 453 articles were screened and considered eligible for further full‐text investigation. The excluded articles were duplicate, reviews, correspondence, editorial, and failed to have inclusion criteria. After analyzing the full texts, only 66 (14.5%, 66 of 453) studies were found to be eligible for further analysis. Based on the inclusion criteria, 45 of 66 (68%) manuscripts were finally selected. Among the selected manuscripts, we extracted epidemiological data from 30 articles, transmission data from 33 articles, clinical symptoms from 18 articles, and treatment and prevention from 9 articles. We also extracted data from two databases, including CDC (USA) and ECDC (Europe) (Figure 1).

### 3.2 Epidemiologic features of 2022‐mpox outbreaks

As of March 29, 2023, a total of 86,716 cases of monkeypox virus had been documented in 110 countries worldwide. Nearly, 99% (85,301 of 86,716) of cases were reported from non‐endemic regions.^1^, ^2^, ^3^, ^5^, ^27^ The highest number of cases has been reported in the Americas (nearly 70%), followed by the EU/EEA (the European Economic Area) (26%).^1^, ^27^ Among the affected countries, the highest numbers of cases have been documented in the United States (30,286), followed by Brazil (10,599), Spain (7505), France (4114), Colombia (4049), United Kingdom (3730), Mexico (3696), and Peru (3695), respectively. The number of mortalities is also prevalent in the United States, followed by Brazil and Peru.^1^, ^2^, ^3^, ^5^, ^27^

The demographic characterization of a large number of cases (29,465) reported to CDC, WHO, and ECDC showed that men (28, 075) were the most infected sex followed by women (864) and transgender women (248), respectively.^1^, ^2^, ^3^, ^5^, ^27^ Further, from the age distribution of the reported cases, we documented that people aged 26–35 years (12,428) had the highest frequency of infection, actually majority of the cases (25,889) was reported in people aged 21–50 years.^1^, ^2^, ^3^, ^5^, ^9^, ^11^, ^13^, ^14^, ^17^, ^19^, ^23^, ^24^, ^25^, ^26^, ^27^ Another study, in 2022, involving 528 patients also reported 100% cases in men with median age 38 years.

Higher number of cases among the White people was reported from the beginning of the outbreaks in May 2022 through 2023, with time the number of Latino and Black or African American increased.^1^, ^2^, ^3^, ^27^ Data from CDC supported that from June 2022 to January 2023, the frequency of Black or African American increased gradually from 30% to 50% of the reported cases. Unusually, the incidence of mpox was reported higher in men (70%–98%) having homosexual and bisexual behaviors in most of the articles and databases (Table 1).^1^, ^2^, ^3^, ^9^, ^10^, ^11^, ^12^, ^13^, ^14^, ^15^, ^16^, ^17^, ^18^, ^21^, ^23^, ^24^, ^26^, ^27^, ^28^, ^29^ Prevalence of notable previous HIV (30%–50%) and sexually transmitted infections (STIs) (20%–50%) among the mpox cases was also documented. Majority of the studies and cases reported to CDC, WHO, and ECDC have also identified HIV as the primary pre‐existing health conditions.^1^, ^2^, ^3^, ^9^, ^11^, ^13^, ^15^, ^16^, ^17^, ^18^, ^21^, ^23^, ^24^, ^26^, ^27^, ^28^, ^29^ The epidemiological curve of the first wave of mpox pandemic has been visualized. One peak with the highest number of confirmed cases reaching 1000 cases/day was apparently confined within a period of 4 months, July 2022 to October 2022. However, mpox‐associated deaths continued with same pace during July 2022 to February 2023.^1^, ^2^, ^3^, ^9^, ^10^, ^11^, ^12^, ^13^, ^14^, ^15^, ^16^, ^17^, ^18^, ^21^, ^22^, ^23^, ^24^, ^25^, ^26^, ^27^, ^28^, ^29^ The highest number of deaths in a single day was recorded in November 10, 2022 (*n* = 6) followed by December 24, 2022 (*n* = 5) and January 14, 2023 (n = 4).^1^, ^2^, ^3^, ^9^, ^10^, ^11^, ^12^, ^13^, ^14^, ^15^, ^16^, ^17^, ^18^, ^21^, ^22^, ^23^, ^24^, ^25^, ^26^, ^27^, ^28^, ^29^

**Table 1:** Epidemiological characteristics of patients with monkeypox virus infection.

				Specific behaviors									
Study	Participants	HIV‐positive	STIs	MSM (%)	Sexual partners, median (IQR)	Probable source of infection	Vaccination	Age, median (IQR)	Race/Ethnicity	Skin^a^	Anogenital	Semen	Saliva
France (Palich et al.)^8^	50	22/50 (44%)	N/A	98%	5 (2–10)	Sex with men	12%	34 (29–40)	White	88%	71%	54%	NA
Spain (Hernaez et al.)^9^	44	23/44 (52%)	27%	94%	Multiple	Sex with men	25%	35 (11.3)	White	N/A	N/A	N/A	85%
Italy (Agrati et al.)^10^	17	7/17 (41%)	35%	100	Multiple	Sex with men, Household	6%	39.5 (33.5–45.25)	White	100%	85	N/A	N/A
France (Mailhe et al.)^11^	264	73/256 (29%)	89%	95%	5 (2–10)	Sex with men	12%	35 (30–41)	White, Black	98%	N/A	N/A	N/A
Spain (Tarín‐Vicente et al.)^12^	181	72/181 (40%)	17%	92%	2.0 (1.0–5.0)	Sexual, Household, Travel, Attendance at a Pride event	18%	37 (31–42)	White, Black	99%	78%	N/A	N/A
Italy (Raccagni et al.)^13^	36	15/36 (42%)	100%	100%	>10	Sex with men	N/A	34 (29–36.5)	White	100%^b^	.	61%	N/A
15 countries (Angelo et al.)^c^, ^14^	226	92/209 (44%)	15%	99%	3 (1–8)	sexual or close intimate contact, Household	16%	37 (32–43)	White	100%	N/A	N/A	N/A
Spain (Peiró‐Mestres et al.)^15^	12	4/12 (33%)	75%	98%	10	sexual or close intimate contact	33%	38.5 (32–52)^d^	White	100%	92%	78%	100%
16 countries (Thornhill et al.)^e^, ^16^	528	218/528 (41%)	109 of 377 (29%)	98%	5 (3–15)	Sexual close contact, Household	9%	38 (18–69)^d^	White, Black	100%^f^	N/A	91%	N/A
Italy (Lapa et al.)^17^	1	1/1 (100%)	100%	100%	5	Sexual close contact,	100%	39	White	NA	N/A	100%	N/A
Germany (Hoffmann et al.)^18^	546	256/546 (47%)	52.4%	100%	Multiple	Sexual contact, travel	12.8%	39 (20–67)^d^	White, Black	100%^f^	N/A	N/A	N/A
Spain (Vivancos‐Gallego et al.)^19^	25	25/25 (100%)	96%	100%	Multiple	Sexual contact, travel	20%	39.5 (33–46)	White	91%	90%	N/A	N/A
Portugal (Duque et al.)^20^	27	14/27 (52%)	52%	18/19 (95%)	Multiple	Sexual contact, travel	4%	33 (22–51)	White	100%^f^	N/A	N/A	N/A
Spain (Echevarría et al.)^21^	49	15/49 (31%)	20%	96%	Multiple	Sexual contact, travel	N/A	37.6^g^	White	100%^f^	80%	N/A	N/A
USA (Philpott et al.)^22^	2891	136/334 (41%)	N/A	94%	Multiple	Sexual contact, travel	48/339 (14%)	35 (30–41)	White, Hispanic, Black	100%^f^	N/A	N/A	N/A
Spain (Iñigo Martínez et al.)^23^	508	225/508 (42%)	N/A	93%	Multiple	Sexual contact, travel, household	N/A	35 (18–67)^d^	White, Black or mixed, Asian	100%^f^	N/A	N/A	N/A
UK (Girometti et al.)^24^	54	13/54 (24%)	25%	100%	Multiple	Sexual contact	N/A	41 (34–45)	White, Black or mixed, Asian	100%^f^	N/A	N/A	N/A
Spain (Orviz et al.)^25^	48	19/48 (40%)	25%	87.5%	Multiple	Sexual contact	25%	35 (29–44)	White	100%^f^	N/A	N/A	N/A
UK (Patel et al.)^26^	197	70/195 (35.9%)	31.5%	99.5%	Multiple	Sexual contact	N/A	38	White, Black or mixed	100%^f^	N/A	N/A	N/A
ECDC (2023)^27^	25,558	3 927/10 366 (38%)	N/A	96%	N/A	Sexual contact	N/A	31–40^d^	White, Black or mixed	100%^f^	N/A	N/A	N/A
Belgium (Hens et al.)^28^	155	34.2	N/A	95.5%	2 (1.0–5.0)	Sexual contact	18%	39.0 (33.0–46.0)	White, Black or mixed	95.6%	96.2%	1/1 (100%)	2/5 (40.0%)
Peru (Maldonado et al.)^29^	205	66%	37.6%	94%	Multiple	Sexual contact, travel		32 (28–38)	Black or mixed	100%	100%	N/A	N/A

Abbreviations: IQR, interquartile range; MSM, men who have sex with men; STI, sexually transmitted infection.

^a^ Includes perianal skin.

^b^ Either skin, anogenital, or oropharyngeal samples combined.

^c^ Argentina, Belgium, Canada, Denmark, France, Germany, Israel, Portugal, South Africa, Spain, Sweden, Romania, The Netherlands, United Kingdom, and United States.

^d^ Range in years.

^e^ Argentina, Australia, Belgium, Canada, Denmark, France, Germany, Israel, Italy, Mexico, Portugal, Spain, Switzerland, The Netherlands, United Kingdom, and United States.

^f^ Skin or anogenital samples combined.

^g^ Mean age.

Compared to the epidemiologic characteristics of the last five decades, we observed unusual features of 2022‐mpox outbreaks.^9^, ^11^, ^13^, ^15^, ^16^, ^17^, ^18^, ^21^, ^23^, ^25^, ^26^, ^27^, ^28^, ^29^, ^33^, ^34^ The spread of non‐travelers’ cases in non‐endemic regions and the large number of cases in a single outbreak are the most unique characteristics of ongoing outbreaks.^23^, ^24^, ^25^, ^26^, ^27^, ^28^, ^29^, ^33^, ^34^ The case fatality rate has also reduced significantly (below 1%) in this 2022 mpox pandemic.^1^, ^2^, ^3^ Further, in mode of transmission we have noticed majority of the study identified involvement of sexual activities in cases of human‐to‐human transmission (Table 1). Another significant unusual characteristic was the higher frequency (60%–100%) of cases identified among men who have sex with men (MSM) and HIV patients.^7^, ^8^, ^9^, ^10^, ^11^, ^12^, ^13^, ^14^, ^15^, ^16^, ^17^, ^18^, ^19^, ^20^, ^21^, ^22^, ^23^, ^24^, ^25^, ^26^, ^27^, ^28^, ^29^ As the pandemic is progressing more studies are required to characterize the epidemiology.

### 3.3 Epidemiologic features of endemic, sporadic cases, and outbreaks

After the first report of mpox case in the Democratic Republic of the Congo (DRC) during 1970s, the outbreaks remained sporadic and endemic in African regions till 2003.^4^, ^33^, ^34^, ^35^, ^36^, ^37^, ^38^, ^39^ During the first three decades, monkeypox virus was documented in DRC, Nigeria, Coˆte d'Ivoire, Cameroon, Liberia, and Sierra Leone. A gradual increase of cases and affected countries continued in the following two decades.^4^, ^33^, ^35^, ^37^, ^39^, ^40^, ^41^, ^42^, ^43^ The endemic cases of monkeypox virus were mostly reported in Central and West African countries, including DRC, Central African Republic (CAR), Republic of the Congo, Cameroon, Gabon, Liberia, Nigeria, Sierra Leone, and South Sudan before the 2022‐mpox outbreak.^4^, ^33^, ^35^, ^37^, ^38^, ^39^, ^42^, ^43^, ^44^, ^45^

After the first report, DRC remained the most affected country with documented confirmed and probable cases of mpox.^4^ After 1970s, the confirmed cases increased in DRC by many folds in every decade.^35^, ^36^, ^37^ Documented confirmed probable or possible mpox cases were 38 during 1970–1979, 343 in 1980–1989, 511 in 1990–1999, and suspected cases were about 10,000 in 2000–2009 and 20,000 in 2010–2019.^4^, ^35^, ^36^, ^37^, ^39^, ^41^, ^43^, ^44^, ^45^ However, due to underreporting a large portion of cases might have not been documented in this surveillance. After DRC, the greatest number of cases have been reported from Nigeria (183 cases) followed by Republic of the Congo (97 cases) and the CAR (69 cases).^4^, ^35^, ^38^, ^39^, ^41^, ^42^, ^43^, ^44^, ^45^

In 2003, monkeypox virus was detected for the first time in the United States and outside Africa.^4^, ^22^, ^46^ About 47 confirmed cases were reported linked to infected Gambian pouched rats from Ghana.^4^, ^22^, ^46^ After that report, no case of monkeypox virus was documented for more than 15 years. Recently in 2018, the first case of monkeypox virus in the United Kingdom was documented followed by one case in Israel in 2018, one case in 2019 in Singapore, three cases in United Kingdom, and one case in the United States in 2021.^1^, ^2^, ^3^, ^4^, ^22^, ^46^, ^47^, ^48^, ^49^, ^50^ The documented cases outside Africa after 2017 were travellers’ cases linked with the outbreaks of mpox in Nigeria during 2017 to 2022.^47^, ^48^, ^49^ Starting in the last quarter of 2017, the outbreak continued through 2022 and 558 confirmed cases were documented with a case fatality ratio of 3.5% from 32 states of Nigeria.^4^, ^47^, ^48^, ^49^ Probable under‐reporting of monkeypox virus has occurred during 2020 to 2022 due to COVID‐19 pandemic.

### 3.4 Clade distribution of monkeypox virus

Three distinct clades of monkeypox virus have been defined, namely I, IIa, and IIb by Happi et al.^51^ Two accepted nomenclatures of genome of monkeypox virus had been reported, namely hmpxv1 focusing on the human‐to‐human transmissible recent isolates after 2017 outbreaks and monkeypox virus including broad diversity of viruses from the discovery till now.^51^, ^52^, ^53^ In the phylogenetic analysis of monkeypox virus, it was documented that isolates from clade IIb were associated with 2022‐outbreak. The tree included 700 genome and MPXV‐M5312_HM12_Rivers was used as the reference sequence. Calculated average divergence of clade IIb was between 0.0015 and 0.0020 (Figure 2A).^53^, ^54^, ^55^

Figure 2(A) Phylogenetic tree of monkeypox virus, (B) distribution of different lineages of hmpxv1 across different continents, (C) divergence of hmpxv1 during the 2022 outbreaks, (D) proportionate distribution of different lineages of hmpxv1 in different regions during the 2022 outbreaks. The trees were built by the maximum composite likelihood method. Reference sequence MPXV‐M5312_HM12_Rivers was used. Data were retrieved from NCBI and trees were adopted from Nextstrain.^54^, ^55^

**Figure:**
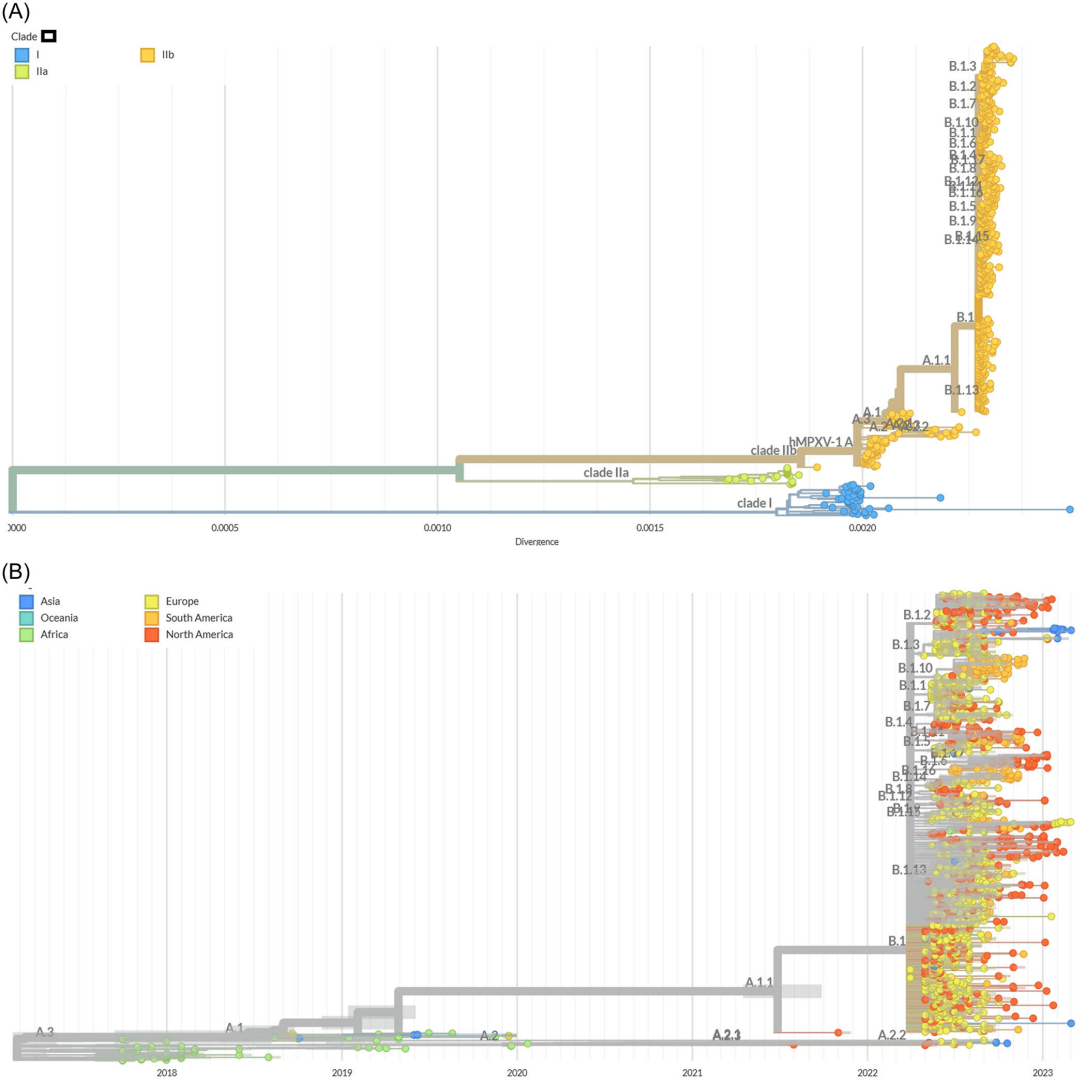

**Figure:**
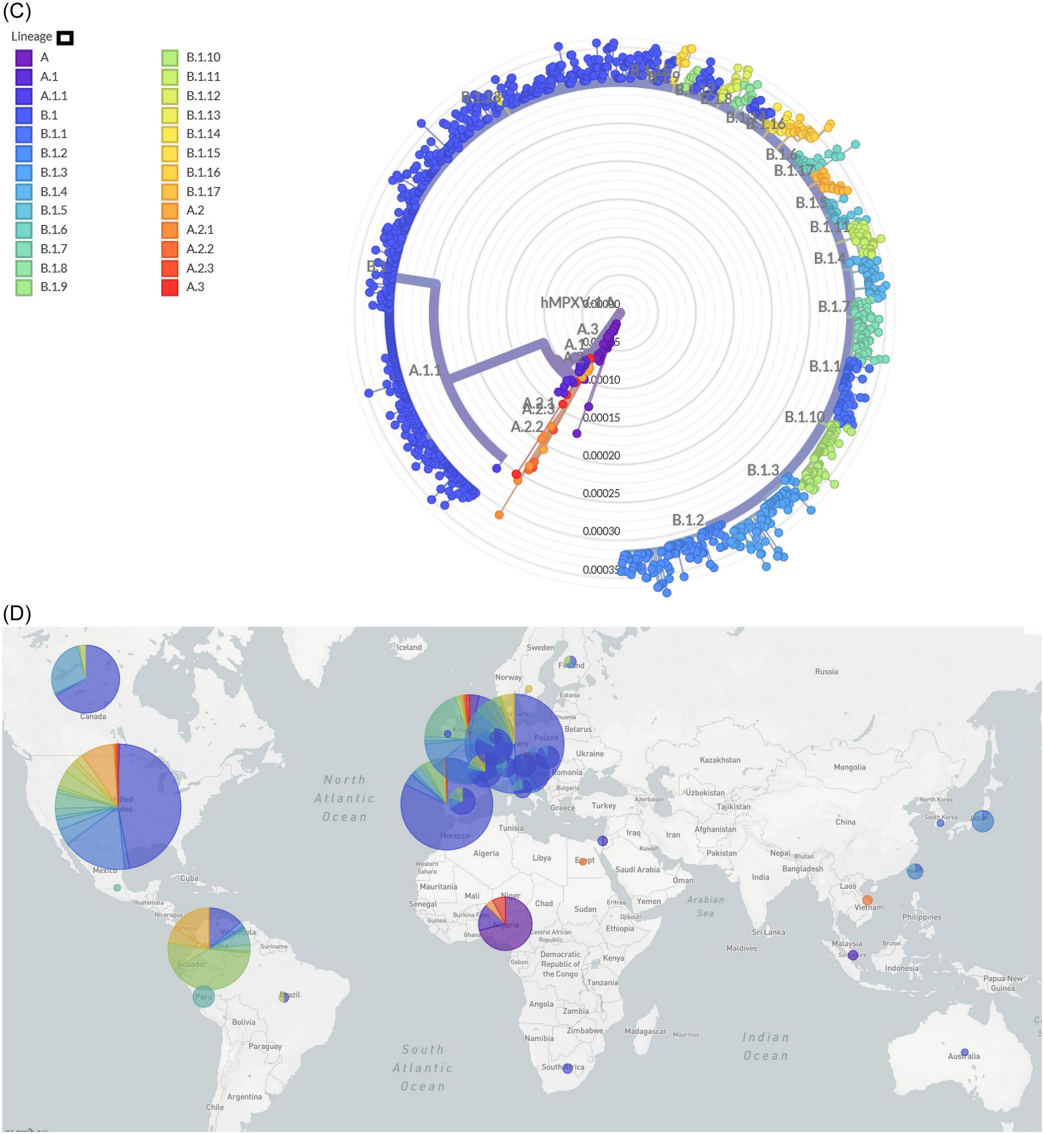

Data from about 1400 whole genome were included for hmpxv1. All of the whole‐genome sequences of hmpxv1 are from the clade IIa. About 27 lineages have been identified based on the genetic divergence till December 2023.^52^, ^53^, ^54^, ^55^ Predominance of lineage B.1 (604 of 1400) is evident followed by B.1.2 (94 of 1400), B.1.10 (71 of 1400), B.1.1 (63 of 1400), B.1.7 (61 of 1400), B.1.3 (46 of 1400), A (44 of 1400), B.1.4 (39 of 1400), B.1.11 (30 of 1400), and B.1.17 (30 of 1400), respectively (Figure 2B). The tree was built by using the maximum composite likelihood model and MPXV‐M5312_HM12_Rivers was used as the reference sequence. From the phylogenetic tree we can see that isolates from lineage B.1 and subsequent lineages were more common in Europe, North America, and South America during 2017 to March 2023. After the 2022‐mpox outbreak, the spread of isolates from lineage B.1 and related lineages including B.1.2, B.1.10, B.1.1, and B.1.3 occurred rapidly in Europe and North America (Figure 2C,D). These data may represent partial diversity as the sequencing of large number of cases is ongoing. Recently evolving lineages of hmpxv1 have an estimated evolutionary rate of ~6 × 10^−5^ subs per site per year. The highest divergence that was calculated for the circulation lineages was 0.00035. The ongoing pandemic isolates of B.1 lineage and associated segregated lineages have a strong evolutionary relationship with the isolates of 2017–2019 outbreaks in Nigeria.^52^, ^53^, ^54^, ^55^ The isolates of B.1 lineage and their descendants have evolved and acquired successful human‐to‐human transmission capability (Table 2). Though the natural history of 2022 mpox pandemic is not well understood, it has several characteristic features than the previous mpox endemic cases, outbreaks, and sporadic cases (Table 1). The most notable epidemiological features of newly evolved isolates include a defined risk group, namely MSM, sexual transmission, and increased median age than previous outbreaks. The pandemic lineages have probably undergone genetic evolution before the onset of the first case of the ongoing pandemic in the first quarter of 2022 and acquired more changes in the genome through human‐to‐human transmission. Extensive studies are required to determine the relationship of evolutionary changes with the newly identified pandemic characteristics.

**Table 2:** Distribution of clades of monkeypox virus along with major epidemiological features.^4^, ^49^, ^50^, ^51^, ^52^, ^53^, ^54^, ^55^, ^56^

Clade nomenclature based on origin	Revised clade	Lineages (*N*)	Reported year	Regions	Known transmission mode	Epidemiologic features	Number of cases
Central Africa	I	0	Early years of report (1970s) to 2020	Congo, CAR, DRC (majority of cases), Gabon, South Sudan, Cameroon	Mainly animal to human, unknown, and human to human	Sporadic cases, outbreaks, and endemic cases Case fatality rate 9.8%	1090 About 30,000^a^
West Africa	IIa	0	Early years of report (1970s) to 2022	Nigeria, Liberia, Sierra Leone, Ivory Coast, Cameroon	Animal to human, human to human, and unknown	Sporadic cases, outbreaks, endemic, and travelers’ cases Case fatality rate 3.5%	252
New West African	IIb	27	2017 to present	Americas, Europe, Africa, Asia, and Oceania	Human to human	Pandemic Case fatality rate 0.1%	85,800

^a^ Suspected cases.

### 3.5 Transmission of mpox virus during outbreaks

Transmission of mpox virus has been occurring since 1960s in the nonhuman primates. Evidence of animal‐to‐animal transmission both in the natural environment and laboratory is well documented.^4^, ^57^, ^58^, ^59^, ^60^ The natural history of mpox virus is yet to be understood well. In the endemic areas, the zoonotic transmission was the most prominent source of human infection (Table 3). However, over time the evidence of animal‐to‐human and human‐to‐human transmission increased in Africa. Close contact with the lesions and body fluids of diseased animals during different activities like deforestation, hunting, slaughtering, and butchering contributed to interspecies transmission of animal‐to‐human infection. Further, animals like rodents get infected when they come in contact with lesions or body fluids of diseased humans. However, virological confirmation is lacking in Central and West African countries on the interspecies transmission of mpox virus.

**Table 3:** Host range, transmission history to humans, and isolation source of mpox virus.^4^, ^34^, ^49^, ^50^, ^51^, ^52^, ^53^, ^54^, ^55^, ^56^, ^57^, ^60^, ^61^, ^62^, ^63^, ^64^, ^65^, ^66^

Species	Family	Related with human infection	Detection	Year	Country	Lineage	Divergence
*Homo sapiens* (humans) *Pan troglodytes* (chimpanzees) *Pongo pygmaeus* (orangutans)	Hominidae/Primates	Yes	Virus isolation from body sit/fluid	1970s–2023 2017 2018	110 countries Côte d'Ivoire	I, IIa, IIb IIa	3.036e^−3^ 1.708e^−3^
*Cercocebus atys* (sooty mangabeys) *Macaca fascicularis* (cynomolgus monkeys)	Cercopithecidae/Primates	No Yes	Virus isolation from body sit/fluid	1958 1964 1965 1959 1962	Denmark Netharlands USA	N/A	N/A
*Callithrix jacchus*	Callithrichidae/Primates	No	Lab. infection	2012	Côte d'Ivoire	IIa	1.655e^−3^
*Oryctolagus cuniculus* (rabbits)	Chinchillidae/Rodentia	No	Lab. infection	1973	Netharlands	N/A	N/A
Hamsters	Cricetidae/Rodentia	No	Lab. infection	1973	Netharlands	N/A	N/A
*Mus musculus* (inbred mouses)	Muridae/Rodentia	No	Lab. infection	2012	DRC	IIa	1.557e^−3^
*Cricetomys* sp. (giant‐pouched rats)	Nesomyidae/Rodentia	No	Virus isolation from body sit/fluid	2003	USA	IIa	1.725e^−3^
*Crocidura littoralis* (butiaba naked‐tailed shrew)	Soricidae	No	Virus isolation from body sit/fluid	2014	DRC	I	2.411e^−3^
*Funisciurus* sp. (rope squirrels) *Funisciurus anerythrus* *Cynomys ludovicianus* (black‐tailed prairie dogs) *Marmota monax* (woodchucks)	Sciuridae/Rodentia	Yes Yes	Virus isolation from body sit/fluid	2003 2014 2003	USA DRC USA	IIa I IIa	1.730e^−3^ 2.427e^−3^ 1.730e^−3^
*Graphiurus* sp. (African dormices)	Gliridae/Rodentia	No	Virus isolation from body sit/fluid	2003	USA	IIa	1.730e^−3^
*Didelphis marsupialis* (southern opossums) *Monodelphis domestica* (shot‐tailed opossums)	Didelphidae/Didelphimorphia	No	Virus isolation from body sit/fluid	2003	USA	IIa	N/A
*Atelerix* sp. (African hedgehogs)	Erinaceidae/Erinaceomorpha	No	Virus isolation from body sit/fluid	2003	USA	IIa	N/A
*Jaculus* sp. (Jerboas)	Dipodidae/Rodentia	No	Virus isolation from body sit/fluid	2003	USA	IIa	N/A
*Atherurus africanus* (porcupines)	Hystricidae/Rodentia	No	Virus isolation from body sit/fluid	2017	DRC, Nigeria	IIa	N/A
*Myrmecophaga tridactyla* (ant‐eaters)	Macroscelididae/Pilosa	No	Virus isolation from body sit/fluid	2003	USA	IIa	N/A

The cessation of smallpox vaccination since 1980s contributed to the wanning of cross‐protection against monkeypox virus.^4^ Human‐to‐human transmission of monkeypox virus has been reported in both Central and West African countries.^4^, ^48^, ^50^, ^56^, ^57^, ^58^, ^59^, ^60^ Contact with skin lesions and fluids of indexed patients among the family members and healthcare providers was the most attributed risk of transmission of monkeypox virus. Contaminated fomites, bedding, and clothing also contributed to the transmission.^4^, ^13^, ^15^, ^18^, ^56^, ^57^, ^59^, ^60^ Sexual transmission of monkeypox virus before the 2022 outbreak was not prominent. Human‐to‐human transmission in both clade I and clade II was documented before the 2022‐mpox pandemic in the endemic regions of Africa (Table 3). The growth (*R*_0_) was estimated below 1 both for the clade I and clade II in endemic and pandemic cases. Traveler cases were mostly reported from clade II and the virus was carried outside the endemic areas. Limited evidence on transmission chains support smaller clusters (not more than 10) of infection from the indexed patient can occur.

As the ongoing outbreak involves the largest number of human cases, the knowledge of the diversity of transmission continues to evolve. Majority of the studies reported evidence of sexual activities for transmission of monkeypox virus in non‐endemic regions in 2022. The clinical, epidemiological, and virological findings strongly support the sexual transmission of monkeypox virus from indexed patients to susceptible persons. Direct contact with the lesions especially on the skin of the genital, anus, anorectum, and throat of patients during sexual activities contributed to the transmission of monkeypox virus (60–100%) among the people (Table 4).^13^, ^15^, ^17^, ^18^, ^19^, ^23^, ^25^, ^27^, ^28^, ^29^ Transmission of monkeypox virus in people after sexual activities with presymptomatic patients has been documented. However, transmission from asymptomatic patients is not reported yet. Virological data on several studies have documented infectious monkeypox virus in semen in patients. Recent studies have reported a higher prevalence (54%–100%) and concentration of mpox DNA in semen samples of the infected men.^13^, ^15^, ^17^, ^18^, ^19^, ^23^, ^25^, ^27^, ^28^, ^29^ Several studies have reported that isolated DNA of monkeypox virus from the lesions of anogenital skin and semen has the capability to infect cell lines like Vero E6 cells and produce cytopathic effects.^12^, ^13^, ^15^, ^17^, ^19^, ^24^, ^26^, ^27^ However, these are preliminary studies to define and characterize the transmission of monkeypox virus via semen. We have also analyzed several studies reporting early evidence of droplet transmission of monkeypox virus via the respiratory route. Nosocomial infection among the healthcare providers has been documented. Transmission via sharp instruments, piercing, and tattooing have also been documented.^13^, ^15^, ^17^, ^18^, ^19^, ^23^, ^25^, ^27^, ^28^, ^29^, ^50^, ^56^, ^58^, ^60^, ^61^, ^62^ There is limited evidence on the fomite transmission of monkeypox virus via contaminated surfaces and objects. However, the potential of contamination of different household objects by monkeypox virus should be evaluated in detail.

**Table 4:** Identified risk factors of transmission of mpox outbreak.^4^, ^8^, ^9^, ^10^, ^11^, ^12^, ^13^, ^14^, ^15^, ^16^, ^17^, ^18^, ^19^, ^20^, ^21^, ^22^, ^23^, ^24^, ^25^, ^26^, ^27^, ^28^, ^29^, ^30^, ^31^, ^34^, ^50^, ^57^, ^58^, ^59^, ^60^, ^64^, ^65^, ^66^, ^67^, ^68^, ^69^

Risk factors	References
Age	Numerous studies have documented that higher frequency of monkeypox virus infection in people of specific age groups.^4^, ^8^, ^9^, ^11^, ^15^, ^18^, ^19^, ^20^, ^21^, ^22^, ^23^, ^25^, ^28^, ^29^, ^30^, ^31^
Sex	Most of the recent published studies found that men are the major group infected by monkeypox virus during 2022 outbreak.^4^, ^8^, ^9^, ^11^, ^15^, ^18^, ^19^, ^20^, ^21^, ^22^, ^23^, ^25^, ^28^, ^29^, ^30^, ^31^
Ethnicity	People of diverse races were infected during this outbreak. However, white people were affected more in the beginning and with time the frequency of black people also increased.^4^, ^8^, ^9^, ^11^, ^15^, ^18^, ^19^, ^20^, ^21^, ^22^, ^23^, ^25^, ^28^, ^29^, ^30^, ^31^
Zoonotic infection	Infection spreading from animals also contributed earlier in this outbreak.^34^, ^50^, ^51^, ^53^, ^54^, ^55^, ^56^, ^57^, ^58^, ^59^, ^60^, ^61^, ^63^, ^64^, ^66^, ^67^, ^68^, ^69^ Further, reports of human‐to‐animal transmission of monkeypox virus was documented.^64^, ^66^
Nosocomial infection	Number of nosocomial infections has been documented in United Kingdom, Spain, and Italy during the 2022 outbreak.^19^, ^21^, ^23^, ^24^, ^25^
Travelers	Travelers have significantly contributed to the spread of 2022‐mpox outbreak across the globe within short period of time.^19^, ^21^, ^23^, ^24^, ^25^
Human‐to‐human	Human‐to‐human transmission contributed to the majority of the cases in 2022 outbreak.^19^, ^21^, ^23^, ^24^, ^25^
Specific behaviors	Specific behaviors, including having homosexual and bisexual behaviors, were largely attributed to majority of the cases.^11^, ^13^, ^15^, ^19^, ^21^, ^23^, ^24^, ^25^, ^27^, ^28^
Pre‐existing health conditions	Different sexually transmitted diseases, including HIV, were found common among the cases.^11^, ^13^, ^15^, ^19^, ^21^, ^23^, ^24^, ^25^, ^26^, ^27^, ^28^, ^29^, ^34^, ^64^, ^65^, ^66^, ^67^, ^68^, ^69^

Studies have documented the presence of infectious monkeypox virus on lesions and major secretions, including urine, feces, nasal or oral droplets and conjunctival exudates in humans and animals.^21^, ^23^, ^28^, ^29^, ^50^, ^56^, ^58^, ^60^ During 2022‐outbreak the probable route of transmission included direct contact, ingestion, and inhalation. Pre‐2022 mpox outbreaks and sporadic cases have transmitted to humans via bites from infected animals, contact with lesions or fluids and blood of infected organs, and aerosols.^4^, ^50^, ^56^, ^58^, ^60^ No confirmed sexual transmission was reported before 2022 outbreak.^4^ Both animal and person‐to‐person transmission were reported from pre‐2022 outbreaks. However, during 2022 outbreak person‐to‐person transmission became the major source of transmission. It is unlikely that person‐to‐person transmission can maintain the virus in humans for a longer period.

Besides human‐to‐human transmission, monkeypox virus has been reported among a diverse group of animals.^4^, ^60^, ^61^, ^63^, ^64^, ^65^, ^66^ The full host range of monkeypox virus is unknown. Documented cases of monkeypox virus include different animals like Old and New World monkeys, apes, rodents, shrews, pigs, small mammals, and dogs.^50^, ^62^, ^63^, ^64^, ^65^, ^66^ Nonhuman primates in both the wild environment including chimpanzees (*Pan troglodytes*) and monkeys of genera Cercopithecus and captivities including captive gorillas (*Gorilla gorilla*), gibbons (*Hylobates lar*), Asian orangutans (*Pongo pygmaeus*), chimpanzees, marmosets (*Hapale jacchus*), Siamiri and Macaca have been documented with clinical infection of mpox.^50^, ^62^, ^63^, ^64^, ^65^, ^66^ In 2003, a larger outbreak of monkeypox virus started from Gambian giant pouched rats (*Cricetomys* spp.) and transmitted to different animals like North American black‐tailed prairie dogs (*Cynomys ludovicianus*), groundhog/woodchuck (*Marmota monax*), dormice (*Graphiurus* sp.), rope squirrels (*Funisciurus* spp.), jerboa (*Jaculus* sp.), and opossums (*Didelphis marsupialis* and *Monodelphis domestica*) in the United States (Table 3). With greater genetic divergence, isolates of clade I were mainly involved in the infection of nonhuman primates in central African regions. Further, isolates of clade IIa were transmitted to diverse groups of animals in West Africa and outside endemic regions, including Europe and the United States.^4^, ^50^, ^60^, ^61^, ^62^, ^63^, ^64^, ^65^, ^66^ The reservoir of mpox viruses is still undermined. Small mammals or rodents in Africa are thought to be reservoirs. Probably, clade I and clade II are maintained in different animal species.

### 3.6 Clinical manifestations of patients with monkeypox virus associated with 2022 outbreak

Monkeypox virus‐infected patients have reported several characteristic clinical symptoms. Majority of the recent studies have documented symptomatic infections.^9^, ^11^, ^12^, ^14^, ^16^, ^17^, ^19^, ^21^, ^22^, ^23^, ^24^, ^25^, ^26^, ^27^, ^34^, ^67^, ^68^, ^69^, ^70^ Only a limited number of studies have reported asymptomatic cases. Among clinical manifestations, characteristic skin rash was the most common (nearly 100% of the study) followed by fever, headache, lymphadenopathy, and myalgia (Table 5).^8^, ^11^, ^13^, ^15^, ^16^, ^17^, ^18^, ^19^, ^20^, ^21^, ^22^, ^23^, ^24^, ^25^, ^26^, ^27^, ^34^, ^68^, ^69^, ^70^ These symptoms were also common during the pre‐2022 outbreaks and sporadic cases. However, the sites of the characteristic skin rash have changed during the 2022 outbreaks. Majority of the studies have reported genital (40%–100% of the patients), perianal (32%–89% of the patients), anal (30%–90% of the patients) as the most common sites of rash followed by trunk, upper limb, oral and peri‐oral areas among the patients (Table 5). However, pre‐2022 outbreaks did not report about genital, anal, and perianal rash in higher frequency.^4^, ^68^, ^69^, ^70^, ^71^ Among other symptoms, lethargy and pharyngitis were reported in the majority of the studies.^8^, ^9^, ^11^, ^13^, ^15^, ^16^, ^17^, ^18^, ^19^, ^21^, ^24^, ^26^, ^29^ Another newly described symptom among the monkeypox virus cases was proctitis, which was documented in significant frequency among patients during the 2022 outbreak. Hospitalization and intensive care units (ICU) admission were required for only a limited number of patients.^8^, ^9^, ^10^, ^11^, ^12^, ^13^, ^14^, ^15^, ^16^, ^17^, ^18^, ^19^, ^20^, ^21^, ^22^, ^23^, ^24^, ^25^, ^26^, ^27^, ^28^, ^29^ Only two of the studies reported deaths of mpox patients. Compared to the pre‐2022 outbreaks, the number of deaths is less in the 2022 outbreak.^4^, ^8^, ^9^, ^11^, ^13^, ^15^, ^16^, ^17^, ^18^, ^19^, ^21^, ^24^, ^26^, ^29^ Monkeypox virus‐infected patients suffer from various health complications. Among them, bacterial superinfection was mostly reported followed by abscesses, bilateral ocular complications, encephalitis, pneumonia, parapharyngeal abscess, mouth ulcers, corneal ulcers, penile edema, balanitis, sepsis, myocarditis, corneal ulcers, and conjunctivitis (Table 5).^1^, ^2^, ^3^, ^8^, ^9^, ^11^, ^13^, ^15^, ^16^, ^17^, ^18^, ^19^, ^21^, ^24^, ^26^, ^29^

**Table 5:** Clinical characteristics of people with monkeypox virus infection during 2022 outbreak.

Study	Participants	Reported clinical features										
		Rash or skin lesions	Fever	Lymphadenopathy	Myalgia	Headache	Lethargy	Pharyngitis	Proctitis	Site of lesions	Other complications	Hospitalization
France (Palich et al.)^8^	50	100%	52%	54%	38%	46%	54%	16%	32%	Skin, anus, anorectal, anogenital	N/A	N/A
Spain (Hernaez et al.)^9^	44	100%	75%			91%			53%	Anogenital, face, trunk, upper limb, genitals	N/A	1 patient
Italy (Agrati et al.)^10^	17	100%			82%			0	N/A	Anogenital, face, trunk, upper limb, genitals	N/A	N/A
France (Mailhe et al.)^11^	264	100%	68%	69%	42%	35%	N/A	20%	18%	Genital and perianal	36% participants	6%
Spain (Tarín‐Vicente et al.)^12^	181	100%	72%	85%	N/A	53%	43%	36%	25%	Genital, skin, anogenital, oral and perioral	78% participants	2%
Italy (Raccagni et al.)^13^	36	100%				N/A				Genital, skin, anogenital, oral	N/A	N/A
15 countries (Angelo et al.)^a^, ^14^	226	61%	58%	61%	14%	16%	41%	24%	15%	Genital, perianal, and trunk	Severe pain and dysphagia	13%
Spain (Peiró‐Mestres et al.)^15^	12	100%	35%	N/A	50%	8	58%	12%	25%	Genital, perianal, and trunk	N/A	
16 countries (Thornhill et al.)^b^, ^16^	528	95%	62%	56%	31%	27%	41%		14%	Genital, perianal, and trunk	Acute kidney injury, severe pain, mostly for severe anorectal pain	13%
Italy (Lapa et al.)^17^	1	100%	100%	N/A	N/A	100%	100%	N/A	100%	Genital, skin, anogenital, oral and perioral	N/A	100%
Germany (Hoffmann et al.)^18^	546	99.2%	53.2%	42.6%	45%	41%	N/A	N/A	N/A	Genital, anal, oral, perioral, head and neck	N/A	4%
Spain (Vivancos‐Gallego et al.)^19^	25	100%	56	84%	16%	48%	16%	20%	52%	Genital, perianal, trunk, and face	90% participants	0
Portugal (Duque et al.)^20^	27	52%	52%	48%	18%	50%	N/A	25%	24%	Genital, perianal, trunk, and face	N/A	11%
Spain (Echevarría et al.)^21^	49	31%	65%	71%	65%	37%	45%	35%	29%	Genital, perianal, and perioral	Myopericarditis, edema, bacterial superinfection	8%
USA (Philpott et al.)^22^	2 891	1,007/1,007 100%	63%	59%	55%	50.8%	57.1%	N/A	22%	Genital, arm, face, perianal, and perioral	N/A	77/954 (8%)
Spain (Iñigo Martínez et al.)^23^	508	98%	63.8%	61.2%	36.4%	31.9%	46.9%	28.1%	15.9%	Anogenital and perineal	Parapharyngeal abscess, mouth ulcers, bacterial superinfection	3.7%
UK (Girometti et al.)^24^	54	100%	57%	55%	30%	20%	67%	20%	N/A	Anogenital, perianal, and perioral	N/A	9%
Spain (Orviz et al.)^25^	48	93.8%	52.1%	62.5%	52.1%	52.1%	66.6%	12.8%	27.1%	Genitals, anogenital, perianal, and perioral	Encephalitis, pneumonia, corneal ulcers	2%
UK (Patel et al.)^26^	197	100%	61.9%	57.9%	31.5%	24.8%	23.4%	16.8%	36.0%	Genitals, perianal	Penile swelling, bleeding, conjunctivitis, bacterial superinfection	10.2%
ECDC^27^	25 558	15 275/15 995 (96%)	10,830/15,995 (68%)	32.7%	10,830/15,995 (68%)			11.2%	2%	Genitals, anogenital, perianal, and perioral	Vomiting, encephalitis, pneumonia, corneal ulcers	5 deaths 7 ICU admission
Belgium (Hens et al.)^28^	155	93.5%	81.0%	46.5%	42.2%	30.2%	8.6%	11.6%	32.3%	Genital, penis, perianal	Bacterial skin infection and penile edema, paraphimosis	22.6% 10 deaths
Peru (Maldonado et al.)^29^	205	81%	79.0%	54.1%	51.5%	58.1%	60%	38.5%	3%	Anogenital area and trunk	Cutaneous bacterial superinfection, balanitis, necrosis of skin lesion	10.2%

^a^ Argentina, Belgium, Canada, Denmark, France, Germany, Israel, Portugal, South Africa, Spain, Sweden, Romania, The Netherlands, UK, and USA.

^b^ Argentina, Australia, Belgium, Canada, Denmark, France, Germany, Israel, Italy, Mexico, Portugal, Spain, Switzerland, The Netherlands, UK, and USA.

### 3.7 Treatment and prevention of mpox virus infected people

Until now there has been no specific medicine or treatment option available against mpox infection.^72^ Drugs used in the treatment of smallpox (tecovirimat or ST‐246) can be used for special cases of mpox.^72^, ^73^, ^74^, ^75^, ^76^, ^77^, ^78^, ^79^ However, the safety and efficacy of tecovirimat in treating people with monkeypox virus is not well‐studied and the food and drug administration (FDA) and CDC have not approved the drug as a treatment option.^75^, ^76^ In severe cases, only tecovirimat is approved to be used with proper concern of the patients. Only the healthcare provider will decide where to use tecovirimat. Other antivirals, including brincidofovir (Tembexa) and cidofovir (Vistide), have also been used in several conditions of mpox infection. Cidofovir, FDA approved injection‐based antiviral against cytomegalovirus infection.^72^, ^73^, ^74^, ^75^, ^76^ Recently, it has been used to treat patients with monkeypox virus. The specific effects of cidofovir on the outcome of monkeypox virus need to be studied in detail. Cidofovir is also not approved for use in treating mpox by FDA (USA) or CDC. Brincidofovir is an oral formulation of cidofovir. It is already approved for the treatment of smallpox by FDA (USA). However, a limited number of studies have reported the effectiveness of brincidofovir against mpox infection. In animal models, brincidofovir has also provided effectiveness against orthopoxvirus infections also.^74^, ^75^, ^77^, ^78^, ^79^ However, the specific effectiveness and side effects of these antivirals in mpox patients need to be evaluated in more detail.

Termination of smallpox vaccination contributed toward the uprise of monkeypox virus cases. The available smallpox vaccine is successful to provide about 85% protection.^4^, ^73^, ^74^, ^75^ The smallpox vaccine provides good cross‐protection against mpox infection. For the prevention of mpox, vaccines, including JYNNEOSTM and ACAM2000®, are approved and used for pre‐ and post‐prophylaxis in specific patients. JYNNEOSTM is a 2‐dose vaccine.^75^ For vaccinations with JYNNEOSTM, a list of recommendation from CDC, USA should be followed. Another vaccine called Aventis Pasteur Smallpox Vaccine (APSV) is also authorized for emergency purposes where the other two vaccines are unavailable or restricted for use.^73^, ^74^, ^75^

## 4 DISCUSSION

Mpox outbreak during 2022 became one of the major public health threats.^1^, ^2^, ^3^ This systematic review integrated epidemiological, clinical, and transmission data of 2022 outbreak of mpox. We used a structured format of data analysis and provided a real‐world overview of mpox outbreak. Beginning from the early 2022, the cases of monkeypox virus started to increase rapidly.^1^, ^2^, ^3^ This is one of the first systematic reviews reporting human‐to‐human transmission of monkeypox virus involving about 90,000 people in 110 regions. In mpox history, it is the largest known outbreak. Local transmission of mpox involving large number of cases in non‐endemic regions has never been documented before 2022 outbreak. About 99% cases were reported from these non‐endemic regions.^1^, ^2^, ^3^, ^5^ The highest number of cases was reported in the Americas (nearly 70%) and EU/EEA (26%) regions, which is the first time in the history of monkeypox virus. The majority of the cases have been documented in the United States (30,000), followed by Brazil (10,599), Spain (7505), France (4114), and Colombia (4049), respectively. However, before this 2022 outbreak cases of monkeypox virus have never exceeded 50 in these countries.^1^, ^2^, ^3^, ^4^ There are several reasons behind this dramatic outbreak of monkeypox virus in the non‐endemic regions. First of all, waning of mass immunity against smallpox has contributed toward the larger outbreak of monkeypox virus. Vaccines against smallpox are effective in providing protection against monkeypox virus. Second, genetic changes and evolution of monkeypox virus may have contributed to the changing epidemiology. Finally, the unaware acts, including lack of knowledge of the mode of transmission of monkeypox virus, involvement in sexual activities of the infected people without proper protection, and traveling, have also contributed to massive spread of monkeypox virus during 2022 outbreak, which is supported by previous studies.^4^, ^36^, ^37^, ^38^, ^41^, ^46^ This 2022‐mpox outbreak may be a sign of a larger pandemic of mopox in the future. Without proper investigation and epidemiological analysis, it will be difficult to prevent mpox outbreaks in the future.

Majority of the studies have reported characteristic epidemiological pattern of 2022‐mpox outbreak distinguished from sporadic cases and local outbreaks reported during the last 50 years in African countries. The majority of the cases were reported among men (95%).^1^, ^2^, ^3^, ^8^, ^10^, ^13^, ^15^, ^18^, ^25^, ^26^, ^27^, ^28^, ^29^ Further, the age of the infected was also distinct from pre‐2022 outbreaks and cases. Most of the cases were reported in people aged between 30 and 40 years, which is a new characteristic of 2022 outbreak. In previous outbreaks in Central and West African countries most of the cases were reported in people aged below 20 years or among young children.^4^, ^33^, ^34^ Our analysis finds that a continuous increase in the median age of infected people is occurring. During 1970–1989, mpox was primarily reported among children aged 4 to 5 years, during 2000–2009 among people aged 10 years and during 2010–2019 among people aged 21 years.^4^, ^33^, ^36^, ^38^, ^42^, ^45^, ^49^, ^50^ The majority of the deaths associated with monkeypox virus were also reported among children aged below 10 years before 2010. However, after 2010 the death rate has shifted swiftly to people aged greater than 20 years. This study suggests that epidemiological changes in mpox outbreaks have been ongoing with consistent changes since 2010. However, majority of the studies on pre‐2022 outbreaks have documented animals as the primary source of monkeypox virus transmission and human‐to‐human cases were rare. During the 2022‐outbreak we detect that majority of the cases (100%) were human‐to‐human transmitted.^9^, ^11^, ^13^, ^14^, ^15^, ^16^, ^18^, ^21^, ^24^, ^29^ These epidemiological changes are probably the result of genetic changes of the virus and origin of strains with altered properties and changes in human behaviors. Among the methods of transmission, direct contact with lesions, body fluids (pus, blood, tissue secretion, droplets of mouth or nose, semen), nonliving objects contaminated with monkeypox virus and droplets contributed to the majority of the cases.^8^, ^10^, ^12^, ^15^, ^18^, ^21^, ^24^, ^27^ Specifically, MSM got infected from their partner through direct contact during sexual activities. Majority of the cases (70%–98%) were reported by MSM men during 2022‐mpox outbreak. However, none of the pre‐2022 studies have reported about the high prevalence of monkeypox virus among MSM men.^4^, ^33^, ^35^, ^37^, ^38^, ^41^, ^45^, ^46^, ^47^, ^48^, ^49^

Another major characteristic of this outbreak was the presence of previous sexually transmitted diseases, including HIV, syphilis, and gonorrhea among the monkeypox virus cases. About 30%–50% of the cases had previous records of HIV‐positive health conditions. These findings are fully supported by the published original research and systematic articles.^9^, ^11^, ^13^, ^14^, ^15^, ^16^, ^18^, ^21^, ^24^, ^29^ However, studies on the correlation between the presence of HIV and other STIs with monkeypox virus are limited and fail to determine their health impact. Characteristic clinical presentations of monkeypox virus cases in 2022 outbreak were different from previous outbreaks and sporadic cases. The majority of the lesions were unevenly distributed in the genitals, perianal, anogenital, trunk, and forearm regions of the patients.^1^, ^2^, ^3^, ^9^, ^11^, ^13^, ^14^, ^15^, ^16^, ^18^, ^21^, ^24^, ^29^ Further, report of fever (60%–85%), weakness (30%–50%), pharyngitis (15%–40%), and proctitis (20%–40%) were more common during 2022 outbreak than pre‐2022 outbreaks. Majority of the cases were reported in the outpatient ward in this outbreak, which was inpatient for pre‐2022 outbreaks.

This study documented that monkeypox virus from clades I, IIa, and IIb were involved in previous outbreaks depending on the geographic regions. However, during the 2022 outbreak, 100% of the isolates were from clade IIb with divergence of 0.0018–0.0035. About 26 lineages of clade IIb evolved and transmitted during 2022 outbreak. In the United States and Europe, isolates from B.1 lineage were the most predominant, followed by B.1.2 and B.1.10. Monkeypox virus variants from clade IIb were involved in the majority of the cases and responsible for distinct epidemiological features from pre‐2022 outbreaks. As more human‐to‐human transmission occur the changes in the genome accelerated.^4^, ^53^, ^54^, ^55^, ^56^, ^57^ Lineage I and IIa were confined to the majority of the cases in Central Africa and West Africa, respectively. However, emergence of the new West African lineage, IIb, was involved with large number of cases outside Africa and in 2022 outbreak. Before 2022 outbreak, isolates from lineage IIa were capable of infecting a large number of wild animals, laboratory animals, and from animal to humans.^4^, ^46^

Though the natural history of monkeypox virus is not fully known, it is predicted that without the animal host, monkeypox virus cannot continue its infection cycle for a longer period in humans alone. However, isolates from IIb have several distinct epidemiological and clinical characteristics. Newer transmission routes of monkeypox virus have been identified during 2022 outbreak, which contributed to the larger community outbreak.^22^, ^23^, ^24^, ^25^, ^26^, ^27^, ^28^, ^29^, ^50^, ^56^, ^57^, ^58^, ^59^, ^60^ For treatment and prevention, no drug or vaccine is approved against monkeypox virus. However, vaccination against smallpox has a good protective effect against monkeypox virus and can be used in specific situations.

The main strength of this study is the using of bias free approach to integrate the findings. This study included well‐defined terms and broad search strategy on mpox without the limit of language, time, and place. In addition, a wide source of information, including gray literature, was also analyzed. However, this study has few limitations. A lower number of actual cases may be presented due to lack of active and proper surveillance. Lack of studies on asymptomatic cases might have influenced the transmission characterization.

## 5 CONCLUSION

The reduction of immunity against smallpox due to discontinuation of vaccination has contributed to the reappearance of monkeypox virus. Evolution of lineage IIb with great capacity to spread from human‐to‐human has impact on the larger outbreak of mpox in the nonendemic regions. We documented distinct epidemiological, clinical, and transmission properties of monkeypox virus during 2022 outbreak. This study reported that men aged 30–40 years with specific sexual behavior (bisexual or MSM) contributed to majority of the infected people. Furthermore, household transmission and human‐to‐human transmission were two main sources of community outbreak. We also detected that distinct clinical feature included appearance of lesions on the genitals, anogenital, perianal, and trunk were common. This study provided an integrated insight into the epidemiology, clinical features, evolution, and transmission of monkeypox virus.

## AUTHOR CONTRIBUTIONS

**Nadim Sharif**: Conceptualization; data curation; formal analysis; investigation; methodology; resources; software; validation; writing—original draft; writing—review & editing. **Nazmul Sharif**: Data curation; formal analysis; project administration; writing—original draft. **Khalid J. Alzahrani**: Methodology; writing—original draft. **Ibrahim F. Halawani**: Formal analysis; writing—review & editing. **Fuad M. Alzahrani**: Software; writing—review & editing. **Isabel De la Torre Díez**: Software; writing—review & editing. **Vivían Lipari**: Data curation; validation. **Miguel Angel López Flores**: Formal analysis; writing—review & editing. **Anowar Khasru Parvez**: Formal analysis; writing—review & editing. **Shuvra Kanti Dey**: Data curation; resources; supervision; validation.

## CONFLICT OF INTEREST STATEMENT

The authors declare no conflict of interest.

## ETHICS STATEMENT

This study was ethically approved by the Biosafety, Biosecurity & Ethical Committee (BBEC) of Jahangirnagar University.

## TRANSPARENCY STATEMENT

The lead author Nadim Sharif affirms that this manuscript is an honest, accurate, and transparent account of the study being reported; that no important aspects of the study have been omitted; and that any discrepancies from the study as planned (and, if relevant, registered) have been explained.

## Data Availability

All relevant data are available in the manuscript. All authors have read and approved the final version of the manuscript NADIM SHARIF had full access to all of the data in this study and takes complete responsibility for the integrity of the data and the accuracy of the data analysis.
